# Identification of key modules in metabolic syndrome induced by second-generation antipsychotics based on co-expression network analysis

**DOI:** 10.1016/j.csbj.2024.01.003

**Published:** 2024-01-05

**Authors:** Ying Sun, Cuizhen Zhu, Lixuan Huang, Chao Luo, Peijun Ju, Jianhua Chen

**Affiliations:** a Shanghai Mental Health Center, Shanghai Jiao Tong University School of Medicine, Shanghai, China; b Shanghai Key Laboratory of Psychotic Disorders, Shanghai, China; c Affiliated Psychological Hospital of Anhui Medical University, Hefei, China; d Anhui Clinical Center for Mental and Psychological Diseases, Hefei Fourth People's Hospital, Hefei, Anhui, China; e Anhui Mental Health Center, Hefei, Anhui, China; f Shanghai Institute of Traditional Chinese Medicine for Mental Health, Shanghai, China; g Yueyang Hospital of Integrated Chinese and Western Medicine, Shanghai University of Traditional Chinese Medicine, Shanghai, China

**Keywords:** Schizophrenia, Metabolic syndrome, Second-generation antipsychotic, RNA sequencing, WGCNA

## Abstract

**Background:**

Second-generation antipsychotics (SGAs) frequently cause metabolic syndrome (MetS), which raises the risk of heart disease, type 2 diabetes, morbid obesity, atherosclerosis, and hypertension. MetS also impairs cognitive function in patients with schizophrenia. However, the fundamental reasons of MetS caused by SGAs are not yet fully understood. Thus, we aimed to identify potential therapeutic targets for MetS induced by SGAs.

**Methods:**

The serum biochemical parameters and the RNA-sequencing of peripheral blood mononuclear cells were measured in three groups (healthy controls and patients with schizophrenia with and without MetS taking SGAs). The study of the weighted gene co-expression network was utilized to pinpoint modules that were significantly connected to clinical markers.

**Results:**

Statistical analysis showed significant differences in triglyceride and high-density lipoprotein among the three groups. The TNF signaling pathway, TGF-β signaling pathway, fatty acid metabolism, NF-kappa B signaling pathway, MAPK signaling pathway, and Toll-like receptor signaling pathway were the pathways that were primarily enriched in the two unique co-expression network modules that were found. Finally, five specific genes (TNF, CXCL8, IL1B, TIMP1, and ESR1) associated with metabolism and immunity pathways were identified.

**Conclusions:**

This study indicated that SGAs differentially induced MetS of patients with schizophrenia through metabolic and inflammation-related pathways. Therefore, the potential side effects of drugs on inflammatory processes need to be considered when using SGAs for the treatment of schizophrenia.

## Graphical Abstract

**Figure:**
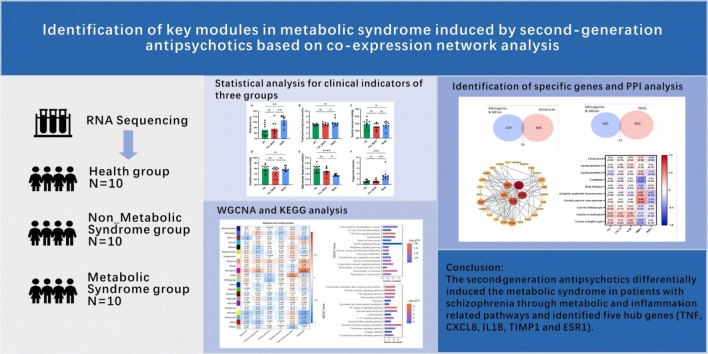

## 1 Introduction

A chronic or recurring mental condition called schizophrenia affects up to 1 % of people worldwide [1]. Hallucinations, paranoia, and disordered thinking are some of its symptoms [2]. In comparison with the general population, those with schizophrenia have a suicide rate that is around 5 % higher, and they also have life expectancies that are 20 years shorter. Second-generation antipsychotics (SGAs) are now widely utilized to treat patients with schizophrenia and have proven to be more effective than first-generation antipsychotics [3]. However, SGAs are likely to induce metabolic syndrome (MetS) in people with schizophrenia. Studies have shown that olanzapine may cause up to 33.4 % of MetS cases [4]. MetS refers to a group of risk factors for cardiovascular disease and, ultimately, premature death in patients with schizophrenia, including insulin resistance, dyslipidemia, high blood pressure, and abdominal obesity [5], [6]. In reality, roughly 20–50 % of individuals with schizophrenia or other psychotic disorders may have their prognosis harmed by medication discontinuation in the long run [7], [8]. Therefore, a solution to the issue of preventing MetS induced by taking SGAs is urgently needed.

SGAs, also referred to as atypical antipsychotics, integrate with serotonin (5-HT), noradrenaline (α, β), and dopamine (D) receptors to effectively treat schizophrenia [9], [10]. Meanwhile, SGAs affect the metabolism of individuals with schizophrenia. They primarily affect the brain, liver, pancreatic beta-cells, adipose tissue, and skeletal muscle, interfering with glucose and lipid homeostasis [11], [12]. For instance, they interact with dopamine, 5-hydroxytryptamine, acetylcholine, and histamine receptors in the hypothalamus, thereby influencing the activity of neuropeptides and 5’AMP-activated protein kinase (AMPK), leading to supraphysiological sympathetic outflow, elevated glucagon levels, and increased hepatic glucose production. Additionally, insulin resistance, dyslipidemia, fat accumulation in the liver and adipose tissue, and altered insulin secretion all contribute to the aggravation of MetS [11]. Prolonged treatment with SGAs also triggers distinct inflammatory cytokine responses in the central nervous system and peripheral adipose tissues, potentially resulting in central chronic inflammatory and immunological abnormalities among patients with schizophrenia [12]. Consequently, comprehensively investigating the mechanisms behind SGA-induced MetS through administration is crucial for personalized and targeted medication use in clinical settings for patients with schizophrenia.

Gene co-expression analysis has yielded deep insights into pathogenesis during the last decade, allowing for the identification of specific genes related to symptoms. As a result, high-throughput sequencing was performed in this work to gather RNA-sequencing data from the peripheral blood of patients with schizophrenia with and without MetS, as well as a healthy control (HC) group. We found modules most associated with metabolic characteristics via weighted gene co-expression network analysis (WGCNA) and then identified specific genes [13], [14]. We investigated the pathways enriched for these genes to find possible mechanisms of action by which SGA differences affect metabolism in patients with schizophrenia.

In this study, we discovered co-expression network modules and five particular genes by the use of RNA sequencing, WGCNA, and protein–protein interaction (PPI) analysis. The results indicated a potential connection between SGA-induced MetS and immune and metabolic pathways. The objective of this study is to provide new targets for the early detection and treatment of MetS caused by SGAs in a clinical setting, as well as to offer a theoretical framework for further investigation into the underlying molecular mechanisms.

## 2 Material & methods

### 2.1 Subjects

**Fig. 1:**
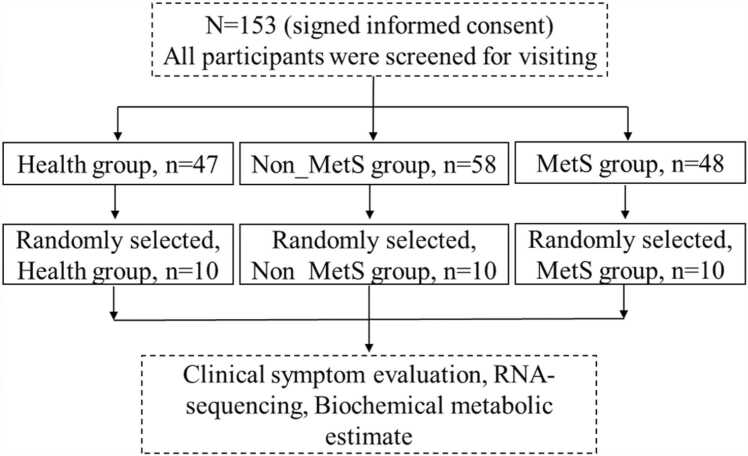
Flow chart.

This research used a cross-sectional design. A total of 106 patients with schizophrenia hospitalized at the Anhui Mental Health Centre (AMHC) between January 2018 and December 2021 and receiving SGA monotherapy were recruited. The 106 patients were split into two groups, namely, MetS (MetS, *n* = 48) and non-MetS (non-MetS, *n* = 58), in accordance with the 2016 Chinese Guidelines for the Management of Dyslipidemia in Adults. The 47 HCs were enlisted by advertising during the same time period from the hospital physical examination center. Finally, 10 samples from each group were chosen at random for RNA sequencing (Fig. 1). The AMHC’s Medical Ethics Committee approved the study. Each participant gave their written informed permission, according to the principles of the Declaration of Helsinki.

The following were the requirements for patients to be included in the MetS group: (1) between the ages of 21 and 57; (2) have received single SGA treatment (olanzapine or risperidone) for longer than 3 months; (3) no current allergies, autoimmune diseases, or infections; (4) no antihyperlipidemic, hypoglycemic, immunosuppressive, and anti-inflammatory medications; (5) meet the DSM-5 criteria for schizophrenia; and (6) meet the criteria for diagnosing MetS in the 2016 Chinese Guidelines for the Management of Dyslipidemia in Adults.

Patients in the non-MetS group had to meet the following criteria to be included: (1) meeting the first to fifth MetS group criteria; and (2) failing to satisfy the 2016 Chinese Guidelines for the Management of Dyslipidemia in Adults.

The inclusion criteria for the healthy controls were as follows: (1) meeting the third and the fourth MetS group criteria; (2) no personal or family history of mental illness; and (3) no history of any neurological disease or traumatic brain injury; The exclusion criteria were as follows: (1) people with severe physical conditions or drug addiction; (2) people with a history of neurological conditions like epilepsy and dementia; (3) people who had recently received electroconvulsive therapy or transcranial magnetic stimulation; and (4) people who were pregnant or nursing.

### 2.2 Definition of MetS

The 2016 Chinese Guidelines for the Management of Dyslipidemia in Adults, which are appropriate for the Chinese population, state that three or more of the following should be present when diagnosing MetS: (1) abdominal obesity, with waist circumference (CM) ≥ 90 for Chinese men and ≥ 85 for Chinese women; (2) a fasting blood glucose (FBG) ≥ 6.1 mmol/L (110 mg/dL) or postprandial 2 h blood glucose ≥ 7.8 mmol/L or a history of diabetes; (3) a blood pressure ≥ 130/85 mmHg and/or a history of hypertension; (4) blood triglyceride (TG) level ≥ 1.7 mmol/L (150 mg/dL); and (5) blood high density lipoprotein (HDL) level < 1.0 mmol/L (40 mg/dL).

### 2.3 Sample collection

After an overnight fast, all patients had their peripheral venous blood samples (5 mL) taken in ethylenediaminetetraacetic acid tubes that had been chilled with ice between 6:30 and 7:00 a.m. Samples were sent right away to the Clinical Laboratory Department. Centrifugation was used to separate the serum at 5 °C. The blood cell pellet was reconstituted in the same volume of PBS. The resulting suspension was gradually placed onto the lymphocyte separation medium inside a centrifuge tube, and centrifugation was performed. The peripheral blood mononuclear cells (PBMCs) within the white layer should be collected into a 1.5 mL Eppendorf tube for centrifugation, and the supernatant should be discarded. An automated biochemical analyzer (AU480, Beckman Counter, USA) was used to measure the levels of FBG, TG, and HDL using a commercial kit (Roche, Switzerland). Prior to blood collection, systolic blood pressure (SBP) and diastolic blood pressure (DBP) were assessed using an automatic sphygmomanometer. At the conclusion of a typical expiration, the waist was measured horizontally across the umbilicus’ center. A sample collection protocol was followed by professional nurses who took all of the samples.

### 2.4 Total RNA extraction and sequencing

The TRIzol reagent manufactured by Invitrogen (Carlsbad, CA, USA) was employed to isolate and purify total RNA. The quantity and quality of total RNA were determined. The high-quality RNA samples with RIN numbers above 7.0 were utilized to construct sequencing libraries. Following total RNA extraction, mRNA was fragmented using the Magnesium RNA Fragmentation Module (NEB, cat. e6150, USA) and heated to 94 °C for a duration of 5–7 min. The RNA fragments were reverse transcribed with SuperScript™ II Reverse Transcriptase (Invitrogen, Cat. No. 1896649, USA) to generate cDNA, which was then used to make a second strand of U-labeled DNA. Each strand of DNA has an A base at the blunt end to enable the ligation process to the indexing adapters. Each adaptor has a T base overhang that enables it to be ligated to the A-tailed DNA segment. After the fragments were ligated together, the size of the double-indexed adapters was determined by employing AMPureXP beads. Following the treatment of the U-labeled two-stranded DNA with heat-stable Uracil-DNA glycosylase (NEB, cat. m0280, USA), PCR was conducted to amplify the ligation products. The average insert size of the resulting cDNA library was 300 ± 50 bp. Finally, we performed 2 × 150 bp paired-end sequencing (PE150) using Illumina Novaseq™ 6000 (LC-Bio Technology Co., Ltd., Hangzhou, China). To reject reads containing aptamers or weak bases, we utilized the Cutadapt tool (version: cutadapt-1.9). FastQC (http://www.bioinformatics.babraham.ac.uk/projects/fastqc/, 0.11.9) was used to check the clean data’s Q20, Q30, and GC content.

We utilized HISAT2 (version: hisat2–2.0.4) software tool to align the data from all samples with the human reference genome. StringTie (version 1.3.4d) was then used to build mapped readings for each sample. To create a comprehensive transcriptome, we combined the transcriptomes of all samples by using the gffcompare tool (version: gffcompare-0.9.8). Following the completion of the transcriptome, the expression level of each transcript was examined using StringTie and ballgown, and the expression of mRNA was determined by calculating the number of transcript fragments per kilobase per million mapped reads (FPKM).

### 2.5 WGCNA of RNA sequencing data

WGCNA was performed based on FPKM expression data using the WGCNA package in R [13]. Genes that had variance values higher than 25 % were all removed and grouped into highly expressed gene branches. A tree-cutting algorithm was used to identify the modules. Abdominal obesity, GLU, SBP, DBP, TG, and HDL were among the trait data that served as diagnostic markers for MetS, and they were utilized to determine module trait correlations. The *org.Hs.eg.db* annotation package was used for the functional annotation of relevant modules. Using the *clusterProfiler* R package, GO enrichment and KEGG enrichment analyses were carried out [15].

### 2.6 Known targets of olanzapine and risperidone

We used the GeneCards database (https://www.genecards.org, version 5.0) to determine the known gene targets of olanzapine and risperidone. Genecards is a comprehensive searchable gene database that integrates the resources of about 150 gene-centric databases, including all functional data for annotating and predicting human genes, as well as genomics, transcriptomics, and proteomics data [16]. Olanzapine and risperidone were entered as keywords to screen relevant gene targets.

### 2.7 PPI network construction and analysis

We constructed the PPI network for overlapping targets between modules and Genecards, as well as genes with statistical differences among the three groups. To obtain PPI networks with rich PPI data gathered from open experimental sources and computational prediction data, we used the STRING database (https://string-db.org, version 11.0) [17]. Each interaction’s confidence level was represented by a score, which ranged from 0 to 1. Shared genes were imported. The raw PPI network (with confidence greater than 0.4) was downloaded, uploaded to Cytoscape 3.8.0, and analyzed according to the topological feature “degree.” We extracted central targets sorted by the degree value and visualized the PPI network of these central targets from the main PPI network.

### 2.8 Statistical analysis

We used GraphPad Prism 9 software (Graphpad Software, San Diego, CA, USA) to analyze and plot data. The results were presented as mean ± standard error of means (SEM). The Shapiro–Wilk test was used to determine whether the distribution of the variables was normal. The independent samples t-test was used to analyze normally distributed variables. The Kruskal–Wallis test was used to analyze variables that were non-normally distributed. Dunnett’s test for multiple comparisons involving more than two groups was used after the ANOVA and Kruskal-Wallis test. Differential genes among the three groups were discriminated using the R package of *edgeR*. Correlation analysis was based on simple linear regression. On the basis of the two-tailed test, statistical significance was set at P < 0.05.

## 3 Results

### 3.1 General demographic background and biochemical results

**Fig. 2:**
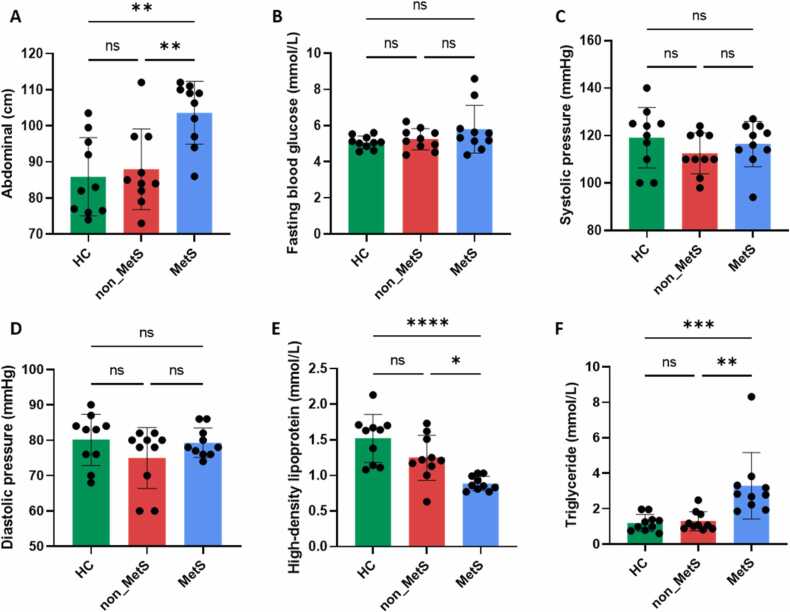
Results of statistical analysis for clinical indicators of three groups. Including abdominal level (A), fasting blood glucose level (B), systolic blood pressure level (C), diastolic blood pressure level (D), high-density lipoprotein level (E), triglyceride level (F). The data are shown as mean ± SEM. *P < 0.05, **P < 0.01, ***P < 0.001, ****P < 0.0001.

Overall, we found no discernible variations in the three groups’ demographic background, such as age and education (P ≥ 0.05; Table 1). Fig. 1 presents the results of relevant indicators for defining MetS. A comparison of the MetS group with the HC and non-MetS groups revealed that the MetS group had considerably greater levels of abdominal fat and TG. Additionally, compared with the HC and non-MetS groups, the MetS group had considerably lower HDL levels. FBG, SBP, and DBP did not significantly differ across the three groups (Fig. 2).

**Table 1:** General demographic background.

Factor	Control (n = 10)	non-MetS (n = 10)	MetS (n = 10)	F(2,27)/T	*P*
Age(year)	37 ± 4.22	35.8 ± 3.43	41.5 ± 4.00	0.59	0.56
Education(year)	4 ± 0.39	3.7 ± 0.21	3 ± 0.33	2.53	0.10
Occupation(none/have)	7/3	2/8	3/7	-	-
Live with family(Y/N)	8/2	3/7	4/6	-	-
PANSS total score	-	58 ± 2.62	57.4 ± 3.18	-	0.89

### 3.2 WGCNA identifies the co-expression module

To further investigate the molecular factors contributing to MetS in patients with schizophrenia who were treated with SGAs, RNA-sequencing was conducted on PBMCs from the three groups: HC, non-MetS group, and MetS group. The samples produced a total of 50.71–55.92 million raw reads. About 38.27–51.14 million clean reads remained after low-quality readings were removed. Approximately 93.9 % of clean reads were successfully mapped to the reference genome per sample (Supplementary file 1). Ultimately, 29,705–34,325 transcripts were obtained, and FPKM values were calculated.

**Fig. 3:**
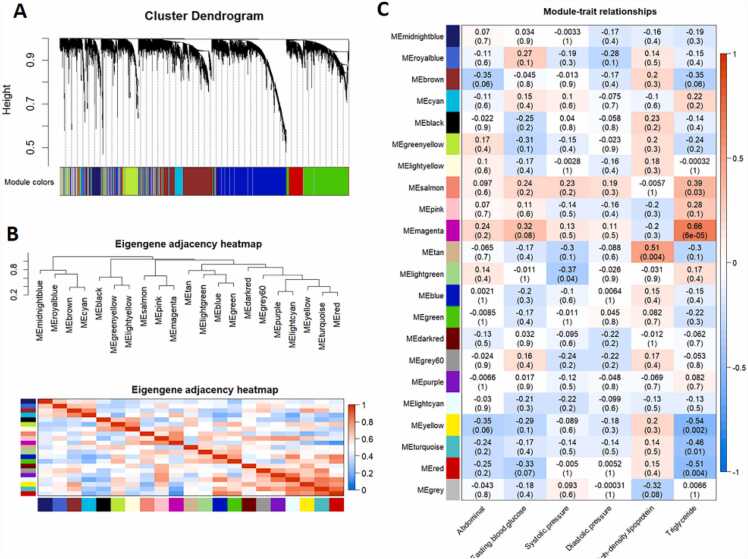
Weighted gene co-expression network analysis (WGCNA). (A) Average connectivity analysis for different soft threshold powers. (B) Heatmap of correlations among WGCNA modules. (C) Heatmap of correlations between sample traits and WGCNA module eigengene. Upper values in each table cell indicate correlations. P-values for association testing are the lower values in brackets.

To investigate the gene expression patterns associated with diagnostic indicators of MetS, we employed WGCNA using the FPKM of RNA-sequencing data. From the transcriptome dataset, 19,072 genes (Supplementary file 2) were grouped based on their co-expression networks. A SoftThreshold (beta) value of 7 was calculated for the construction of co-expression networks. Topological overlap matrices were clustered using average association hierarchy. Highly connected gene branches were produced, clipped, and colored. Twenty modules (MEmidnightblue, MEroyablue, MEbrown, MEcyan, MEgreenyellow, MElightyellow, MEsalmon, MEpink, MEmagenta, MEtan, MElightgreen, MEblue, MEgreen, MEdarkred, MEgrey60, MEpurple, MElightcyan, MEyellow, MEturquoise, and MEred) were identified (Fig. 3A and B). Notably, the MEgrey module contained unassigned genes. Module Eigengene (ME) functioned as representatives of the modules, and the correlation of ME among modules was calculated (Fig. 3C). Using the WGCNA package in R, we further assessed the relationship between each ME and sample traits including abdominal measurements, FBG, SBP, DBP, HDL, and TG levels. The MEmagenta module showed the best association with TG levels among the 20 modules (R = 0.66, p = 0.00006), whereas the MEtan module demonstrated the highest correlation with HDL levels (R = 0.51, p = 0.004).

### 3.3 Analysis of the MEmagenta module

**Fig. 4:**
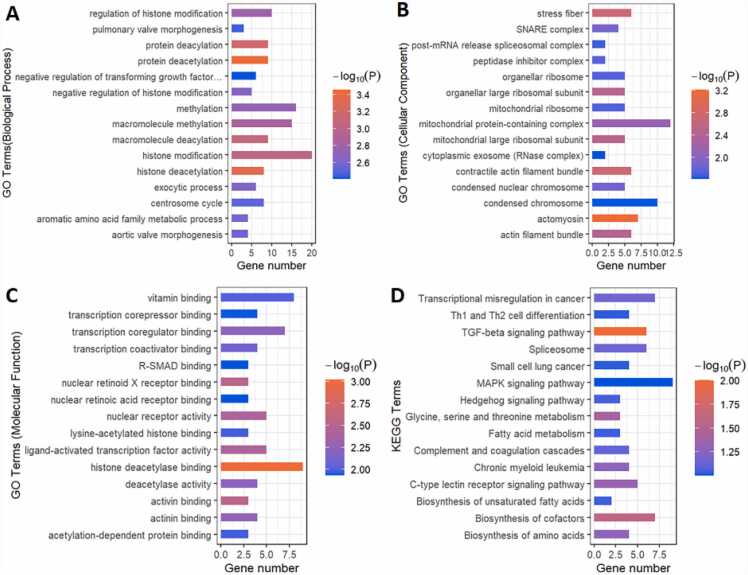
Gene Analysis for the MEmagenta module genes. Top 15 GO terms related to biological processes (A), cellular component (B) and molecular function (C) of MEmagenta module. (D) KEGG enrichment analysis of MEmagenta module. Terms are arranged by P value. The number of gene types is represented by the gene number.

On the MEmagenta module, we performed GO and KEGG enrichment analyses. The biological process, cellular component, and molecular function comprised the three parts of GO enrichment analysis (Fig. 4A–C; Supplementary file 3). The top 15 GO keywords were chosen based on their P-values. The module genes provided information about potential biological processes such as protein deacetylation and histone modification. The cellular components included actomyosin and stress fiber. In terms of molecular function, the module genes showed associations with functions such as histone deacetylase binding and nuclear retinoid X receptor binding. The higher-level systemic functions that were possibly involved were also identified by the KEGG enrichment analysis of module genes, including the TGF signaling pathway; glycine, serine, and threonine metabolism; Hedgehog signaling pathway; biosynthesis of unsaturated fatty acids, fatty acid metabolism; and MAPK signaling pathway (Supplemental file 4, Fig. 4D).

### 3.4 Analysis of the MEtan module

**Fig. 5:**
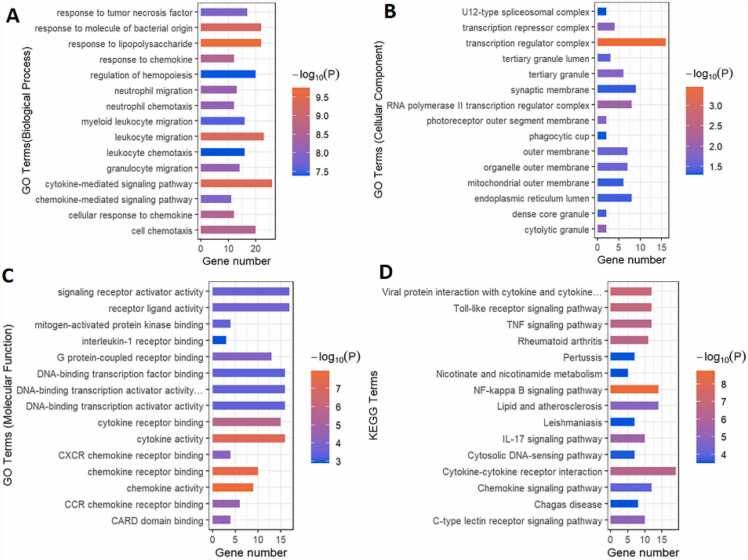
Analysis of MEtan module genes. Top 15 GO terms related to the biological process (A), cellular component (B) and molecular function (C) of MEtan module. (D) KEGG enrichment analysis of MEtan module. Terms are arranged by P value. The number of gene types is represented by the gene number.

On the MEtan module, we also performed GO enrichment and KEGG enrichment studies. Fig. 4 shows the top 15 GO and KEGG terms sorted by their P-values. The analysis of the genes of the MEtan module revealed potential biological processes including response to lipopolysaccharide and cytokine-mediated signaling pathway. As for cellular components, the module genes were found to be related to transcription regulator complexes, RNA polymerase II transcription regulator complexes, and other components. In terms of molecular functions, the module showed associations with chemokine activity, cytokine activity, and other functions (Fig. 5A–C, Supplementary file 5). On the genes in the MEtan module, we also ran KEGG enrichment analysis. The findings showed a notable enrichment in a number of pathways, including the NF-kappa B signaling pathway, Toll-like receptor (TLR) signaling pathway, TNF signaling pathway, and cytokine–cytokine receptor interaction (Fig. 5D, Supplementary file 6).

### 3.5 Analysis of specific genes

**Fig. 6:**
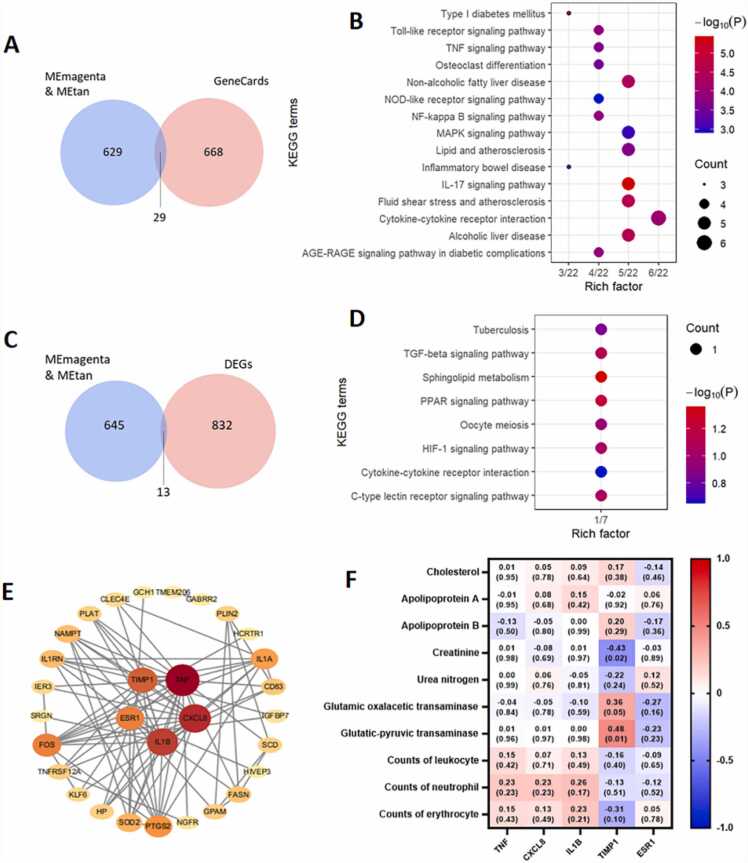
Identification and analysis of specific genes. (A) Venn diagram showing the genes from Genecards and two modules (MEmagenta and MEtan). (B) The top 15 terms of KEGG enrichment analysis sorted by the P value for overlapping genes in modules and Genecards. The number of gene types is represented by the gene number. (C) Venn diagram of genes of two modules (MEmagenta and MEtan) and genes with statistically different among the three groups. (D) The top 15 terms of KEGG enrichment analysis sorted by the P value for overlapping genes in modules and differential genes among the three groups. Gene number represents the number of gene types. (E) The protein–protein interaction (PPI) network of the 42 overlapping genes. The node color and size represent the magnitude of the degree value. (F) The association analysis of the 5 genes and the measured blood indexes. Upper values in each table cell indicate correlations. P-values for association testing are the lower values in brackets.

To identify specific genes within the MEmagenta and MEtan modules, we obtained 697 known gene targets of olanzapine/risperidone from the Genecards database (Supplementary file 7). We found 29 overlapping genes between these targets and the genes within the modules (Fig. 6A).

The KEGG enrichment analysis of these 29 genes revealed enrichment in pathways such as the IL-17 signaling pathway, NF-kappa B signaling pathway, and TLR signaling pathway (Fig. 6B). Of the 845 genes that showed statistical differences among the three groups (Supplementary file 8), 13 genes were shared with the genes within the modules. These common genes were largely abundant in the TGF signaling pathway and sphingolipid metabolism (Fig. 6C and D). Primarily using STRING online, a PPI network of the 42 overlapping genes was created and uploaded to Cytoscape 3.7.1. Initially, the network consisted of 30 nodes and 88 edges when considering interactions with a confidence score over 0.400. We then extracted the top five targets (TNF, CXCL8, IL1B, TIMP1, and ESR1) based on their degree values within the network (Fig. 6E). In addition, we measured various blood indexes including cholesterol, apolipoprotein A, apolipoprotein B, creatinine, urea nitrogen, glutamic oxalacetic transaminase, and glutamic–pyruvic transaminase in the three groups. The data are listed in the supplementary materials (Supplementary Fig. 1). TIMP1 was significantly correlated with glutamic–pyruvic transaminase (R = 0.48, P = 0.01) and creatinine (R = −0.43, P = 0.02; Fig. 6F) in an association analysis between the 5 genes and the observed blood indices.

## 4 Discussion

MetS induced by SGA, a prevalent clinical phenomenon, has garnered significant attention from researchers regarding the mechanism behind its development [18], [19]. Thus, our study collected PBMCs from patients with schizophrenia taking olanzapine or risperidone that induced and did not induce MetS and then sequenced them for RNA. Utilizing WGCNA, we identified two highly significant modules associated with TG and HDL levels.

TG is a crucial indicator in evaluating MetS, and the associated module genes primarily participate in lipid-related metabolic pathways. These pathways include the TGF-β signaling pathway; Hedgehog signaling pathway; glycine, serine, and threonine metabolism; biosynthesis of unsaturated fatty acids; fatty acid metabolism; and MAPK signaling pathway. TGF-β1–3, activins/inhibins, growth differentiation factors, myostatin, and BMPs are all members of the TGF superfamily. They have a variety of functions in controlling glucose homeostasis, lipid metabolism, and hunger. Studies have shown that the TGF signaling pathway is highly involved in the emergence of obesity. By encouraging white fat browning and boosting mitochondrial biogenesis, limiting or blocking this pathway may prevent obesity [20], [21], [22]. In metabolic illnesses linked to obesity, type 2 diabetes, and non-alcoholic fatty liver disease, circulation levels of glycine, an amino acid that is considered to be conditionally necessary, are frequently found to be low [23]. Adipose tissue formation is inhibited by the Hedgehog signaling system. Hedgehog signaling activation prevents high-fat diet-induced obesity, improves systemic glucose tolerance, and increases insulin sensitivity [24]. By serving as nutrients or having an immediate impact on the hypothalamus, unsaturated fatty acids have the potential to mitigate the negative impacts of obesity. They can lower body fat and counteract diet-induced inflammation [25], [26]. In obese people, plasma free fatty acid levels are elevated, which leads to insulin resistance in the liver, endothelial cells, skeletal muscle, and other important insulin target organs. They play an important role in the connection among atherosclerotic vascular disease, MetS, and obesity [26], [27]. Studies have shown that the adipocyte-level regulation of lipolysis by insulin involves hypothalamic MAPK activation. The inhibitory effect of brain insulin signaling on lipolysis is lessened in mice when the MAPK pathway is blocked, but not the PI3K pathway [28].

The “vascular scavenger,” or HDL, carries cholesterol from the surrounding tissues, which is then changed into bile acids or eliminated straight from the intestine via bile. It is a plasma lipoprotein that prevents atherosclerosis and serves as a safeguard against coronary heart disease. Previous research has shown that HDL has a role in innate immunity and has anti-inflammatory properties even in the absence of acute or ongoing inflammation [29], [30]. The module genes that show the strongest correlation with HDL are primarily enriched in pathways associated with metabolism and inflammation. NF-kappa B signaling pathway, TLR signaling pathway, TNF signaling pathway, and cytokine–cytokine receptor interaction are a few of them. The first line of defense against pathogens is composed of TLRs, which are also critical in bridging innate and adaptive immune responses [31]. For instance, TLR3 gene knockout mice exhibit attenuated macrophage infiltration into adipose tissue, accompanied with NF-κB-dependent inhibition of the AMPK/Akt signaling pathway [32]. By modifying metabolic hormones like insulin and glucagon, NF-κB can control metabolic processes [33]. White adipose tissue stresses or lipopolysaccharides increase NF-κB and c-Jun N-terminal kinase signaling, upregulate the production of inflammatory cytokines such as TNF-α and IL-6, and promote insulin resistance in adipocytes and macrophages via TLR4/TLR2 activation [34], [35].

Furthermore, we identified overlapping genes. These overlapping genes were largely enriched in signaling pathways such as the TNF signaling route and the TGF signaling pathway, which corresponded to the findings of the modular gene enrichment analysis. In addition, a number of clinical trials have shown that the administration of olanzapine to patients with schizophrenia causes an increase in plasma cytokines like TNF-α, IL-6, and IL-1 [18], [36]. Therefore, our research implied that mRNA in PBMCs may participate in the regulation of metabolic and inflammatory processes by altering plasma TNF-α levels via the TNF-α and TGF-β signaling pathways. The immune mechanisms related to metabolism might play a critical role in SGA-induced MetS. Additionally, long-term use of SGAs triggers certain immune cell responses in the central nervous system and peripheral adipose tissue, resulting in inflammation and immunological abnormalities. Through PPI analysis, we identified five hub genes (TNF, CXCL8, IL1B, TIMP1, and ESR1), among which the TIMP1 gene exhibited a significant correlation with glutamic–pyruvic transaminase. According to studies, plasma levels of TIMP1 are increased in obesity and related disorders such steatosis, which can lead to the onset of hepatic steatosis and glucose intolerance brought on by food [37]. However, further research is necessary to ascertain TIMP1’s contribution to MetS induction by SGAs.

Notably, this study had some limitations. First, as a cross-sectional study, the sample size was small. Second, diverse ethnic groups could enhance the study’s applicability and validity. Furthermore, the results of the study were based only on sequencing data and lacked experimental validation from basic research. Further validation of the enriched pathways should be prioritized in future studies.

## 5 Conclusions

This study observed significant differences in serum TG and HDL levels between individuals taking SGAs that induce MetS and those not inducing MetS. Through WGCNA, we identified the two most significant modules associated with TG and HDL. The module genes were mainly enriched in the metabolic and inflammation-related pathways. These findings were further supported by published data and three sets of differential genes. Our results indicated that SGAs differentially induced MetS in patients with schizophrenia through metabolic and inflammation-related pathways. Therefore, the potential side effects of drugs on metabolic and inflammatory processes need to be considered when using SGAs for the treatment of schizophrenia.

## Ethics approval and informed consent

The AMHC's Medical Ethics Committee gave the study their blessing. Each participant gave their written informed permission, as per the tenets of the Declaration of Helsinki. The trial has the clinical registration number ChiCTR2100045240.

## Funding

This work was supported by the 10.13039/501100001809National Natural Science Foundation of China (grant number 82071500), the Three-year Action Plan Project of Shanghai Traditional Chinese Medicine Development (grant number ZY-(2021-2023)-0207-01), “Scientific and Technological Innovation Action Plan” of Shanghai Science and Technology Commission of China (grant numbers 21Y11921100, 21XD1423300), Shanghai “Pujiang Talents” Program (grant number 21PJD063), 10.13039/100007219Shanghai Natural Science Foundation (grant number 23ZR1454600), Personnel Training Program of Shanghai Mental Health Center (grant number 2021-QH-04), and Research Open Project of Shanghai Institute of Traditional Chinese Medicine for Mental Health (grant number SZB2023204).

## CRediT authorship contribution statement

YS designed the study, interpreted data, and wrote the manuscript. CZ collected the samples and acquired data. LH and CL contributed to data acquisition. JC and PJ designed this project and revised the manuscript. All authors read and approved the final manuscript.

## Declaration of Competing Interest

None of the authors of this manuscript have any conflicts of interests to report.
