# Supplementary figures and images for: Identification of key modules in metabolic syndrome induced by second-generation antipsychotics based on co-expression network analysis
Source: Comput Struct Biotechnol J. 2024 Jan 5;23:723–31. doi: 10.1016/j.csbj.2024.01.003 (PMC10826125; doi:10.1016/j.csbj.2024.01.003)

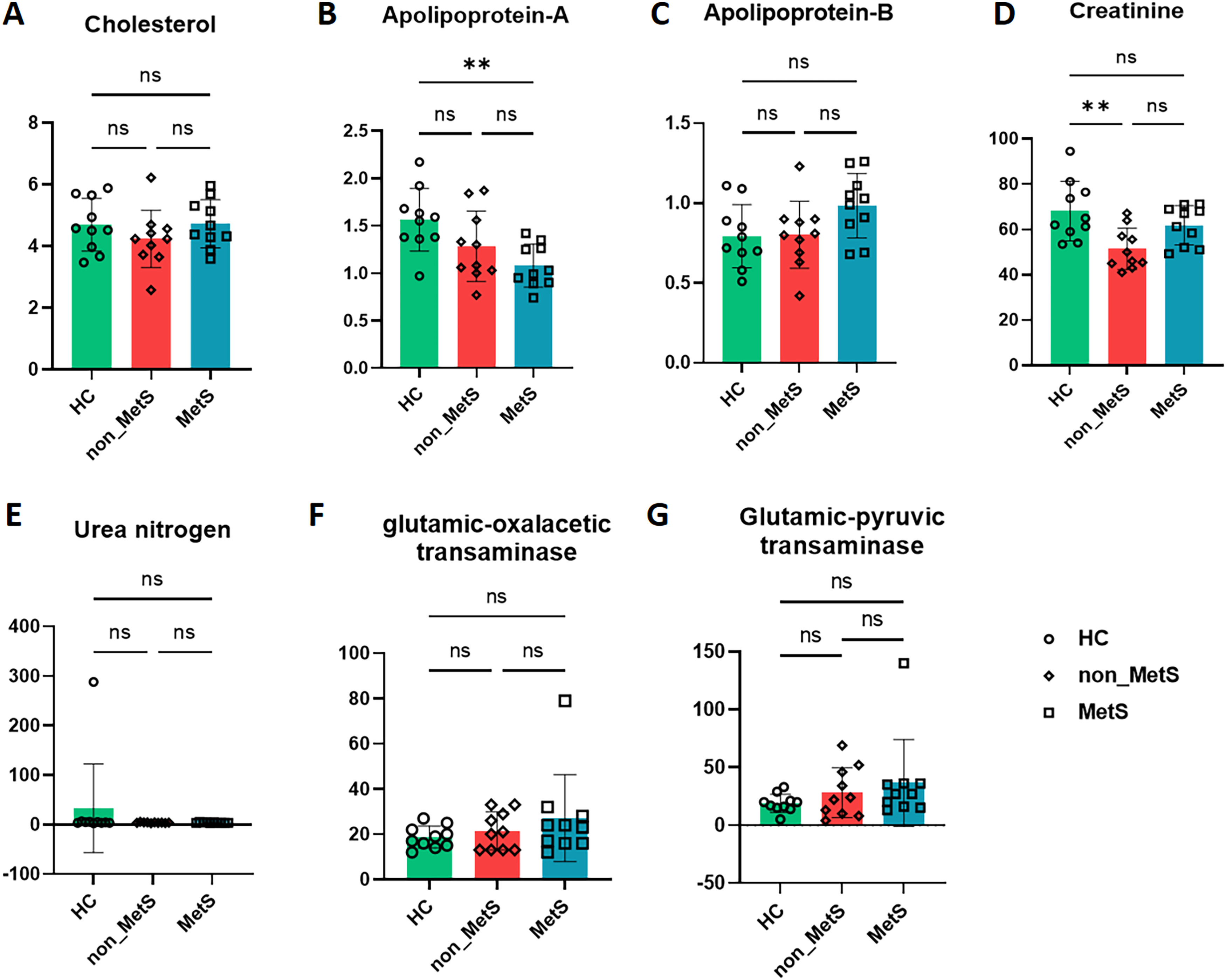

Supplement: Supplementary file 9 — Supplementary material. [file mmc9.jpg]
